# Ensemble stacking of machine learning models for air quality prediction for Hyderabad city in India

**DOI:** 10.1016/j.isci.2025.111894

**Published:** 2025-01-25

**Authors:** Gokulan Ravindiran, K. Karthick, Sivarethinamohan Rajamanickam, Deepshikha Datta, Bimal Das, G. Shyamala, Gasim Hayder, Azees Maria

**Affiliations:** 1 Department of Civil Engineering, Dayananda Sagar College of Engineering, Bengaluru, Karnataka 560078, India; 2 Institute of Energy Infrastructure, Universiti Tenaga Nasional (UNITEN), 43000 Kajang, Selangor Darul Ehsan, Malaysia; 3 Department of Electrical and Electronics Engineering, GMR Institute of Technology, Rajam 532 127, Andhra Pradesh, India; 4 Symbiosis Centre for Management Studies, Bengaluru Campus, Symbiosis International (Deemed University), Bengaluru, Karnataka, India; 5 Department of Chemistry, Brainware University, Barasat, Kolkata, West Bengal, India; 6 Department of Chemical Engineering, National Institute of Technology, Durgapur, Durgapur, West Bengal, India; 7 Department of Civil Engineering, SR University, Warangal 506371, Telangana, India; 8 Department of Civil Engineering, College of Engineering, Universiti Tenaga Nasional (UNITEN), 43000 Kajang, Selangor Darul Ehsan, Malaysia; 9 School of Computer Science and Engineering, VIT-AP University, Amaravati, Andhra Pradesh 522 237, India

**Keywords:** Environmental health, Environmental monitoring, Artificial intelligence

## Abstract

Hyderabad, one of the rapidly developing cities in India, is facing with severe air pollution due to rapid urbanization, industrial operations, and climatic factors. To alleviate the negative impact on human health and the environment, accurate monitoring and forecasting of air quality are essential. This research utilized various machine learning models, such as XGBoost, LarsCV, Bayesian Ridge, AdaBoost, and ensemble stacking methods, to forecast the air quality index (AQI) using data from August 2016 to October 2023, which included 18 different air pollutants, including meteorological parameters. The ensemble stacking method showed excellent performance, attaining high training (R^2^ = 0.994) and validation (R^2^ = 0.999) accuracy with low error metrics (mean absolute error [MAE] = 0.496, mean square error [MSE] = 0.429, root-mean-square error [RMSE] = 0.655). These results highlight the efficacy of ensemble stacking for AQI prediction, providing crucial information for policymakers to formulate strategies to reduce air pollution’s effects on public health and environmental sustainability.

## Highlights

• Air quality index (AQI) is predicted for Hyderabad city, India
• Machine learning models used air pollutants and meteorological parameters to predict AQI
• Hyperparameter tuning was done using GridSearchCV with 5-fold cross-validation
• Ensemble stacking achieves top accuracy with R^2^ of 0.999 and low error rates

## Graphical abstract

**Figure:**
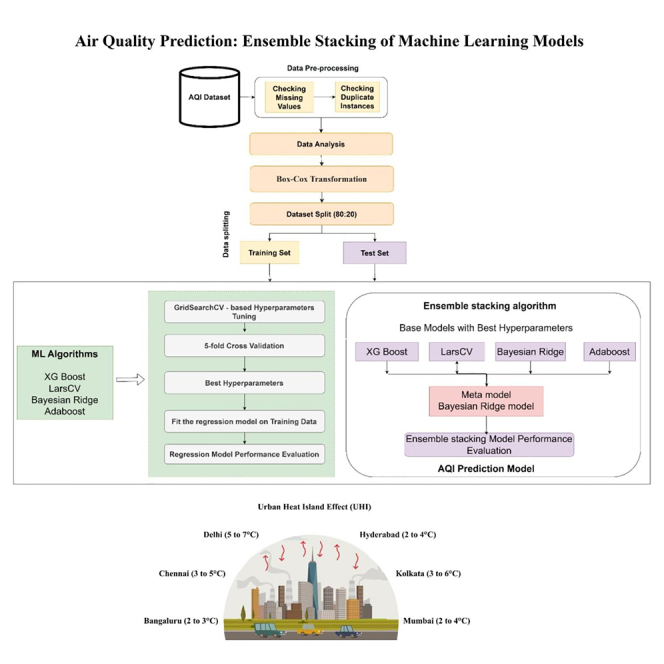

## Introduction

Air pollution is considered one of the most serious threats to humans and the environment compared to all other pollution.^1^ Air pollutants associated with human health disorders include particulate matter 2.5 (PM_2.5_) and 10 (PM_10_), carbon monoxide, ozone, nitrogen dioxide, and sulfur dioxide. The World Health Organization (WHO) reported that approximately 11% of deaths are due to air pollution, and 92% of the population lives in polluted cities. Air pollution is the most common and severe impact in low- and middle-income countries where air pollutant concentration in the atmosphere increases vigorously due to urbanization and industrial activities.^2^ PM_2.5_ exposure is the maximum in Asia, Africa, and the Middle East. Some air pollutants, like black carbon and ozone precursors, play a role in global warming, whereas greenhouse gases (GHGs) are the main factors driving climate change. Many governments and non-governmental organizations (NGOs) are combating the impact of climate change by adopting strict regulations. To successfully implement air pollution control strategies, it is essential to monitor air pollutant emissions, analyze air quality, and predict or forecast air quality.^3^ Almost all countries have started monitoring and recording air pollutants in major cities and using them to adapt and implement strategies to reduce them.^4^

Air pollution is a complex problem, and analyzing the reason for high levels of air pollutants that exist in a region is difficult because several parameters affect the air quality. Metrological factors, namely, rainfall, wind speed, and wind direction, play a major role in the dispersion of air pollutants from one place to another. The city’s geography also plays a major role in the elevated atmospheric concentrations.^5^ India is also a developing country where air pollution is a significant problem.^6^ In India, it is predicted that, by 2050, the urban population will account for approximately 53%.^7^ This urban development will change land use and land cover patterns and will be a root cause of climate change.^8^^,^^9^^,^^10^ Furthermore, urbanization increases anthropogenic emissions, such as carbon dioxide, methane, and nitrous oxide. It also results in natural disasters, such as extreme heat, heat waves, floods, droughts, and changes in rainfall patterns.^11^ Urbanization also increases the emission of aerosols and other GHGs into the atmosphere due to vehicular and industrial emissions.^12^

The 2022 World Air Quality Report focused on PM_2.5_ and examined 7,323 cities in 131 countries, unions, and regions (https://shorturl.at/UjpO1). IQAir is a Swiss air quality monitoring company that releases AQI annually to understand the global trends in many developed and developing countries. IQAir aims to deliver data about the current status of air pollution to the government, NGOs, researchers, educators, and several other organizations. As per the report released by IQAir, only 13 out of 131 countries meet the guideline values of PM_2.5_ less than the 5 μg/m^3^ prescribed by the WHO. In India, the PM_2.5_ concentration was observed as 53.3 μg/m^3^ and ranked eighth; in 2021, the average PM_2.5_ concentration was 58.5 μg/m^3^ and ranked fifth globally. It was observed that, of the 20 top-polluted cities, 14 were from India. Bhiwadi, India, stood at third position in the world and first in India with an average PM_2.5_ concentration of 92.7 μg/m^3^ and 106.2 μg/m^3^ in 2021. It is also observed that all 14 cities are from north India, five are from Bihar (Darbhanga, Asopur, Patna, Chapra, and Muzaffarpur), three towns are from Haryana (Dharuhera, Bahadurgarh, and Faridabad), three cities are from Uttar Pradesh (Ghaziabad, Muzaffarnagar, and Greater Noida), two are from Delhi (Delhi [National Capital Territory] and New Delhi), and one city is from Rajasthan (Bhiwadi). All five states are located very close to each other. Industrial activities, agricultural practices, and alluvial soil along the Gangetic plains are some of the primary causes of pollution. Alluvium, as a fertile soil, is made up of loose, unconsolidated particles, and as a result, dry alluvial soil contributes significantly to wind-blown dust.

The air quality index (AQI) determines the air quality and categorizes it as good, satisfactory, moderate, poor, or very poor. Table S1 summarizes the different AQI categories and sub-indexes of pollutants with possible human health impacts. In India, the AQI is determined according to the standards set by the Central Pollution Control Board (CPCB) as part of the National Air Quality Index framework. For every pollutant, a sub-index is generated using the measured concentration of the pollutant and its relevant breakpoint values outlined in the CPCB AQI table. The overall AQI is calculated based on the highest sub-index value from the pollutants measured. This approach ensures that the pollutant with the most significant health risk influences the AQI. The AQI is divided into six categories (from good to severe), each linked with specific color codes and health recommendations to assist the public in interpreting and reacting to air quality conditions. Forecasting the AQI has numerous significant applications and advantages, especially regarding public health, urban development, and environmental stewardship. The primary benefits include safeguarding health, aiding policy and planning, optimizing resources, promoting climate and ecological gains, providing economic advantages, and enhancing disaster readiness. By utilizing machine learning (ML) and sophisticated predictive models, AQI prediction enables communities, governments, and industries to take proactive measures, leading to improved health outcomes, sustainable growth, and strengthened environmental resilience.

Forecasting AQI is very important, and ML models are promising techniques compared to conventional methods. ML is crucial for enhancing air quality through its sophisticated abilities to monitor, forecast, and regulate air pollution. By utilizing its potential to analyze intricate datasets, reveal underlying trends, and offer practical recommendations, ML dramatically improves the ability to oversee, anticipate, and control air quality, leading to healthier environments and enhanced quality of life.

The present study uses an ensemble stacking algorithm model to forecast the AQI of Hyderabad city and Telangana state of India. In recent years, Hyderabad has experienced several climate change impacts and urban heat island effects.^13^ The current PM_2.5_ concentration in Hyderabad exceeds the WHO annual air quality guideline value by 5.9 times. According to the report released by IQAir 2023, based on PM_2.5_, Hyderabad ranked 308^th^ globally, 301^st^ in the Asia continent, 151^st^ in India, and 2^nd^ in Telangana State.^14^ In the southern part of India, Hyderabad is a rapidly growing metropolitan city that provides a strong basis for forecasting the AQI owing to its elevated pollution levels. Hyderabad faces significant air quality issues caused by industrial operations, vehicle emissions, and construction dust. The city’s air pollution has recently deteriorated, consistently surpassing both the WHO and national standards for PM_2.5_ concentrations, posing serious health risks to its residents. Furthermore, its distinct climate and seasonal variations make predicting AQI crucial to managing public health and policy measures. The city’s high population density and urban expansion further worsen these conditions, emphasizing the necessity for accurate AQI prediction to improve adverse environmental and human health impacts.

## Results and discussion

### AQI

**Figure 1:**
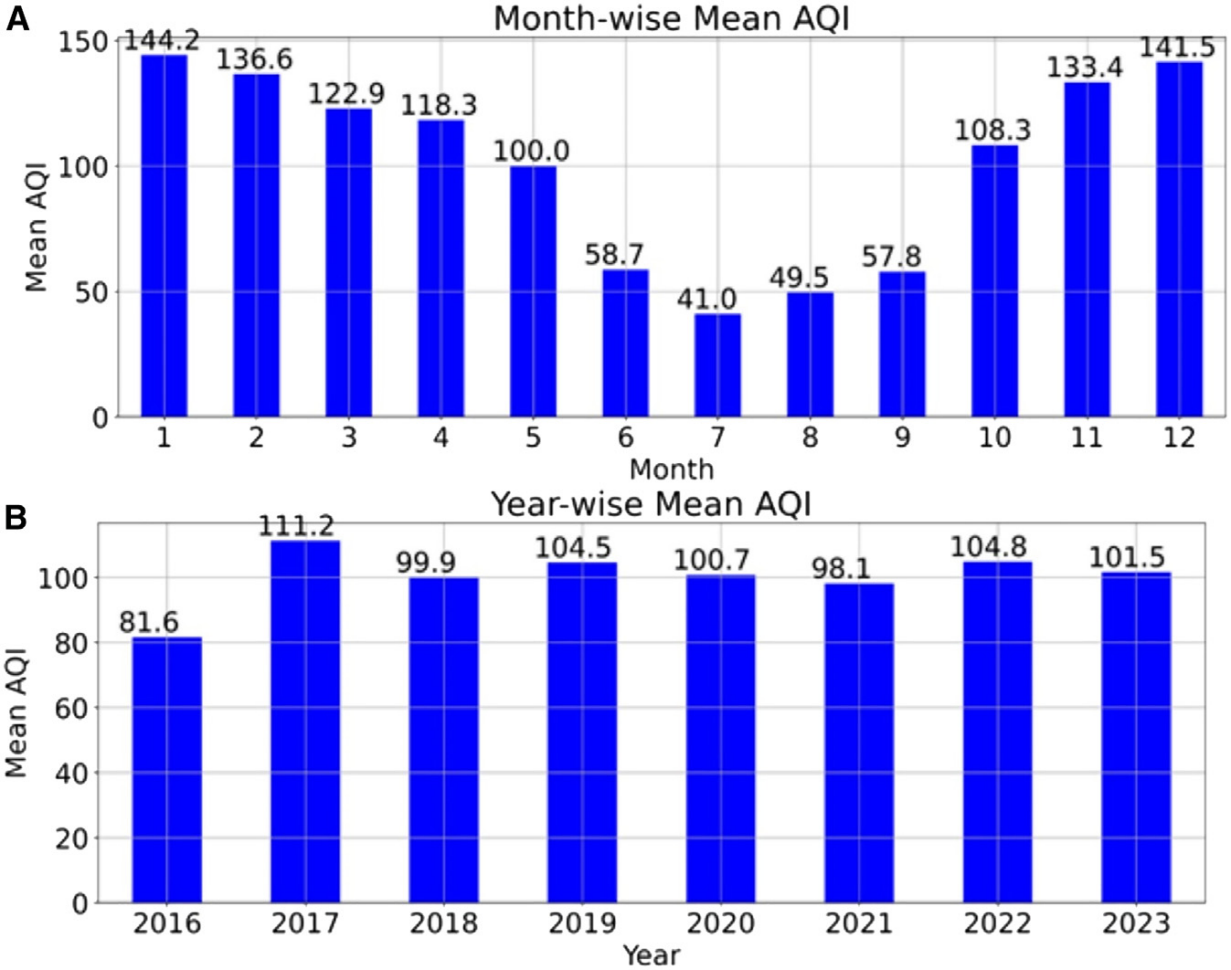
Mean AQI variation of Hyderabad city (A) Monthly variation. (B) Yearly variation. This graph illustrates the monthly and yearly variations of air quality index of Hyderabad city from 2016 to 2023.

Figure 1 illustrates the seasonal and annual variations in the AQI. The maximum mean AQI was observed at 111.2, followed by the minimum AQI in 2016. It is observed that the AQI ranges between 81.6 and 111.2, indicating that the air is moderately polluted and that long-term exposure will result in health issues for human health and affect the environment. The results also clearly demonstrated that AQI was higher in winter than in the summer and monsoon seasons. The winter season in India spans between November and February, and the AQI was 133.4, 14.1.5, 144.2, and 136.6 for November, December, January, and February, respectively. The higher AQI in winter may be due to temperature inversion, slower wind speed, Diwali festival, vehicular emissions, and construction activities.^15^ The AQI also decreased drastically from June to September, and the AQI rate was between 41 and 58.7. The probable reason for the reduced AQI was the monsoon season. During the monsoon period, atmospheric pollutants are collected and dispersed to the surroundings by heavy rainfall and high wind speed.^16^ Figure 1 shows that AQI values decreased from 104.5 in 2019 to 100.4 in 2020 and decreased to 98.1 in 2021. This was due to the COVID-19 pandemic lockdown, where vehicular emissions were reduced drastically and infrastructure development was almost stopped, which might have reduced air pollutants.^17^ Based on seasonal trends, some strategies that could mitigate air pollution include strengthening vehicle emission standards during winter and encouraging using natural gas or electric heating systems.

### PM_2.5_ and PM_10_

**Figure 2:**
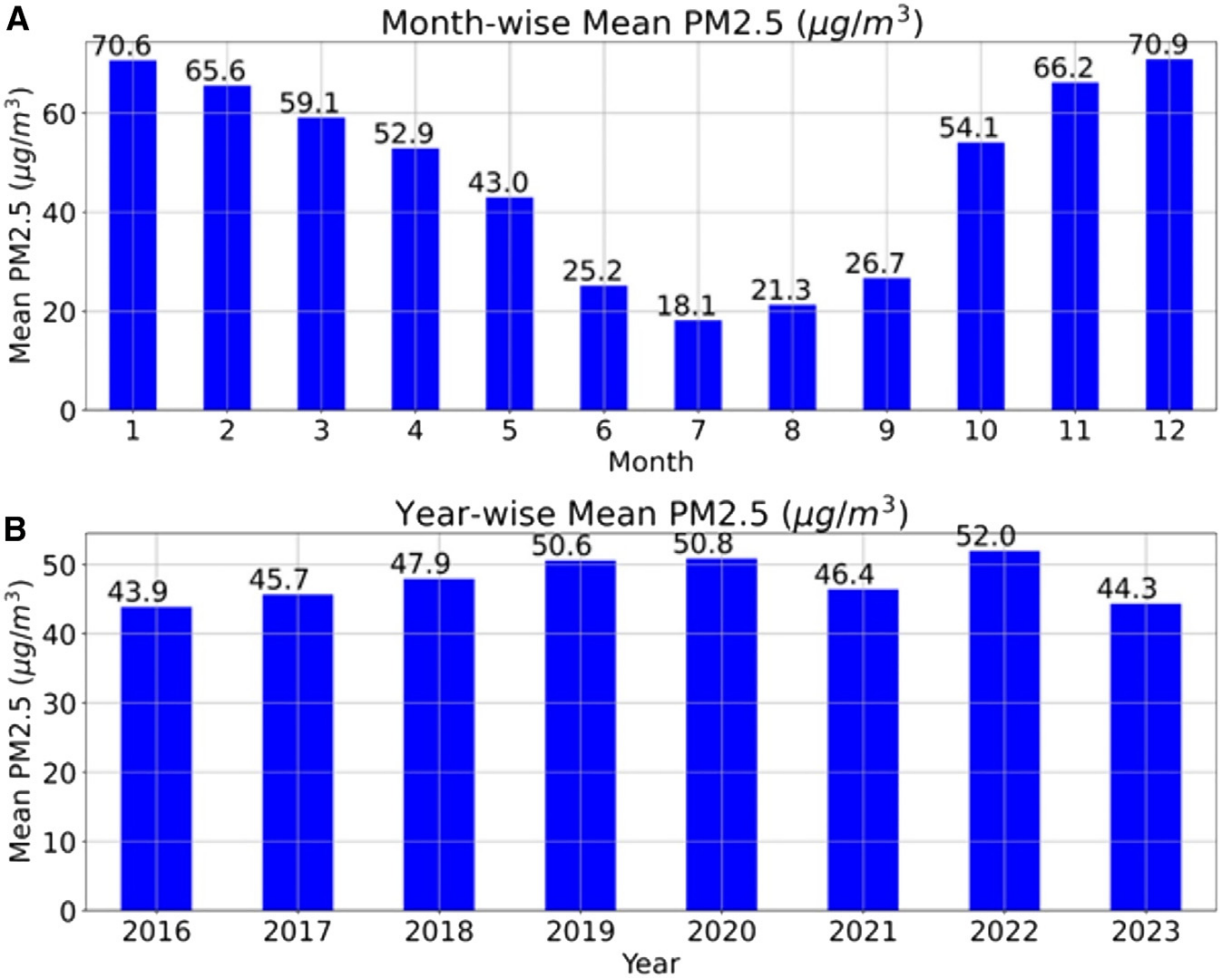
Mean PM_2.5_ variation of Hyderabad city (A) Monthly variation. (B) Yearly variation. This graph illustrates the monthly and yearly variations of particulate matter 2.5 (PM_2.5_) in Hyderabad city, India, from 20016 to 2023.

**Figure 3:**
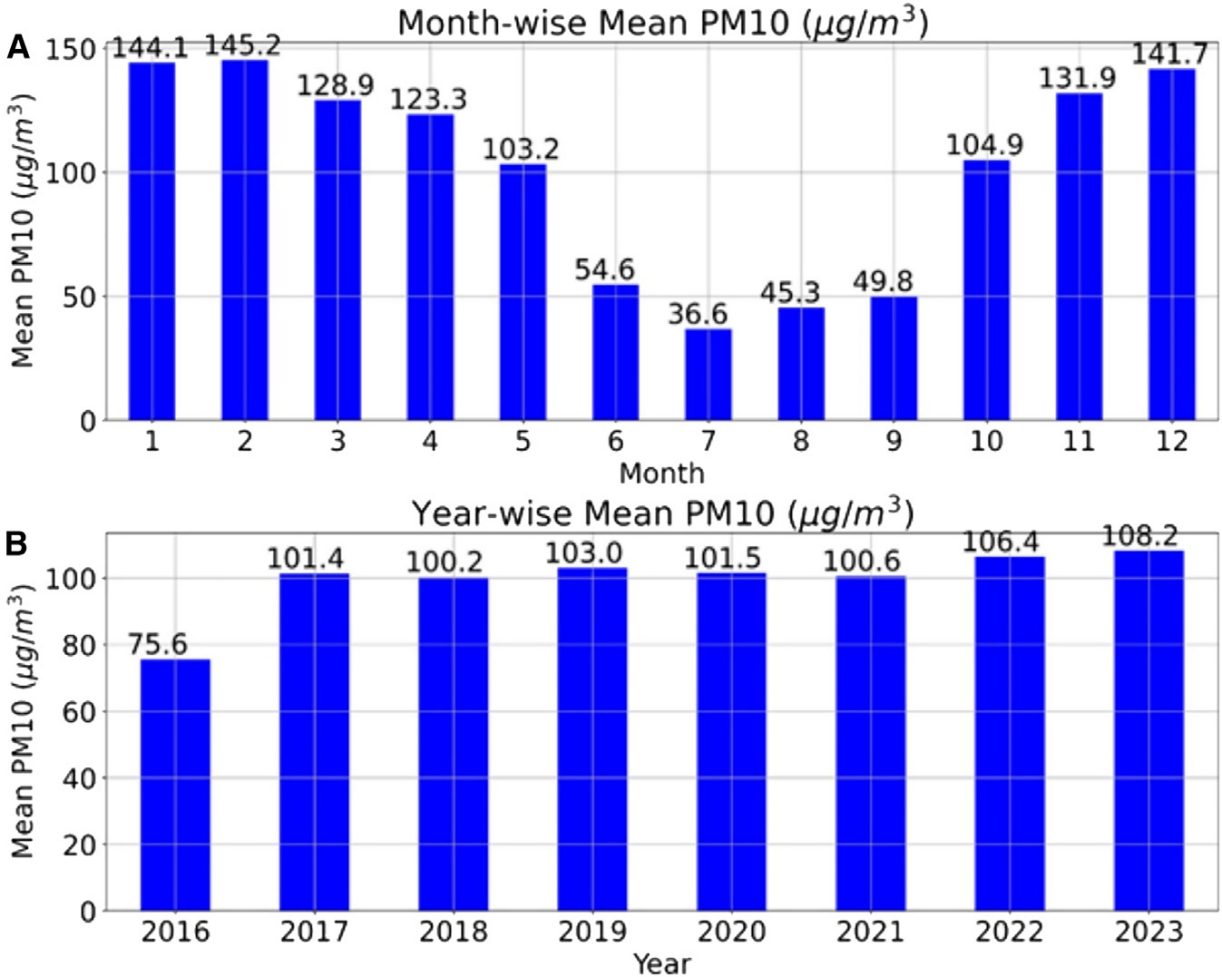
Mean PM_10_ variation of Hyderabad city (A) Monthly variation. (B) Yearly variation. This graph illustrates the monthly and yearly variations of particulate matter 10 (PM_10_) in Hyderabad city, India, from 20016 to 2023.

Figures 2 and 3 illustrate the seasonal and monthly variations in PM_2.5_ and PM_10._ The results show that, for PM_2.5_, the concentration increased from 43.9 to 52 from 2016 to 2022 but suddenly decreased in 2021 and 2023. The decrease in concentration may be due to several reasons, namely the COVID-19 impact in 2021; seasonal and weather conditions; and adapting green initiatives, policies, and regulations. In 2023, the average mean value may be slightly less than that in 2022; this may also be due to the availability of datasets. The datasets were used until October 2023, and, generally, the values were high in November and December, which may also reduce the average mean values. PM_2.5_, in 2021, is lower than that in 2020, despite the COVID-19 lockdown in 2020. This may be due to the pre-existing PM_2.5_ levels being high in 2020, and the lockdown in 2020 might have reduced the available PM_2.5_, so reduced PM_2.5_ was observed in 2021 compared to 2020. It is also observed that the seasonal variation trend of particulate matter is similar to AQI, confirming that PM_2.5_ and PM_10_ play a significant role in the final AQI values. Therefore, it is recommended that green initiatives and technological advancements be initiated to reduce PM_2.5_, PM_10_, and other pollutants to reduce AQI values.

### Gaseous pollutants and meteorological factors

Gaseous pollutants are equally significant in determining the AQI and maintaining a healthy environment. Like PM_2.5_, higher concentrations of gaseous pollutants in the atmosphere harm human health and the environment. The key gaseous pollutants with severe effects were O_3_, NO_2_, SO_2_, CO, and NH_3_. The study datasets revealed that NOx, NO_2_, and CO significantly impacted the AQI values, with correlation coefficients of 0.59, 0.58, and 0.57, respectively. Other gaseous pollutants, namely ozone, benzene, xylene, and toluene, correlate negatively. Figure S2 illustrates the variation in gaseous pollutants, with NO concentration reaching approximately 40 μg/m³ from 2016 to 2019. During 2020, the concentration of NO was maintained in the range between 2 and 2.6 μg/m³, a reduction attributed to the impact of the COVID-19 pandemic. The lockdown led to a drastic reduction in vehicular emissions and limited industrial operations, resulting in lower levels. Post-COVID, the NO concentration started to increase in the atmosphere by 2021. However, the atmospheric concentration was less than that in the pre-COVID situation, possibly due to increased emission standards and other meteorological parameters.^18^ The same trend was observed for NO_2_, NOx, NH_3_, SO_2_, and O_3_. NO_2_ concentrations were high during the period 2016 to 2020, and, after 2020, the NO_2_ concentration also decreased. Initially, the NO_2_ concentration varied between 10 and 100 μg/m³ during 2016–2020, and, after 2020, the NO_2_ concentration was found to be less than 60 μg/m³ and varied between 20 and 40 μg/m³. NO_2_ was found to have strong correlations of 0.60, 0.59, and 0.95 with PM_2.5_, PM_10_, and NOx, respectively.

O_3_ levels increased from 2016 to 2018, reaching a maximum concentration of 120 μg/m³. After 2020, the ozone concentrations were stable with minor fluctuations, and this concentration was much lower than those from 2016 to 2018. A similar trend was observed for the NOx emissions from 2016 to 2018. The NOx concentration was high and gradually decreased until 2020. The NO_x_ concentration was found to stay decreased between 2020 and 2024, and the average concentration was between 40 and 60 μg/m³. NOx emissions mainly come from vehicular emissions, which started reducing in 2020, possibly due to the implementation of Bharat stage VI norms in 2020 and the utilization of electric vehicles.^19^ NO_x_ is considered as one of the main precursors for the formation of ground-level ozone. Because of solar irradiation, nitrogen oxides and volatile organic compounds react and form ground-level ozone. Therefore, increased NO_x_ emissions will result in increased ground-level ozone and vice versa.^20^

Similar to NOx and O_3_, NH_3_ exhibited the same trend. From 2016 to early 2020, the NH_3_ concentration was found to have a maximum concentration of 60–80 μg/m³, and, on most days, it was between 10 and 40 μg/m³ after 2020. The NH_3_ concentration was reduced to be less than 20 μg/m³. No direct link exists between NO_x_ and O_3_, but, due to the COVID-19 lockdown, reduced industrial emissions might have reduced these levels. The main source of NH_3_ emissions is agricultural activities, where the utilization of fertilizers results in the emission of ammonia, and waste management is another primary sector that results in NH_3_ emissions.^21^ The reduced NH_3_ concentration may reduce the utilization of fewer fertilizers in and around Hyderabad city and improve waste management strategies adopted by the government.

NH_3_ is strongly correlated with temperature (0.57) and vertical wind speed (0.60), and an increase in temperature results in ammonia volatilization from the soil, organic manure, and fertilizers. During hot or warm climates, NH_3_ concentrations will be high in the atmosphere because the volatilization rate of ammonia from the sources will be maximum.^22^ Generally, ammonia is highly soluble in water, and an increase in temperature warms water bodies, resulting in a lower solubility. This results in the release of more gaseous ammonia into the atmosphere. Ammonia reacts with other gaseous pollutants, SO_2_ and NO_x_, to form secondary particulate matter.

These gaseous pollutants at elevated levels cause several health issues. Prolonged exposure to NOx elevates the chances of experiencing strokes and heart diseases. Short exposure to ozone leads to respiratory irritation, reduced lung capacity, and worsening asthma symptoms. Long-term exposure to ozone increases the likelihood of developing chronic respiratory problems like chronic obstructive pulmonary disease. Short-term exposure to SOx irritates the respiratory tract and results in coughing and difficulty in breathing, while extended exposure results in lung diseases, such as bronchitis. NH₃ acts as an irritant, leading to coughing and throat irritation. CO interacts with hemoglobin to produce carboxyhemoglobin, which reduces oxygen transport in the bloodstream, resulting in headaches, dizziness, and potentially death at elevated levels. Extended exposure to CO impacts both the cardiovascular and neurological systems. Benzene is considered carcinogenic and is associated with leukemia and various other cancers. Xylene can lead to headaches, dizziness, and neurological impacts with long-term exposure.^1^

Metrological factors also play a significant role in atmospheric pollutant concentrations. Wind speed and direction play significant roles in the dispersion of pollutants, and relative humidity also plays a crucial role in air pollution.^23^ Figure S3 illustrates the different meteorological parameters and their seasonal variation. Temperature, relative humidity, and solar radiation exhibit uniform seasonal variations. Small spikes and fluctuations may be due to extreme weather events like cyclones and storms. Some parameters are strongly correlated, namely, wind speed, which corresponds to a decrease in barometric pressure, and relative humidity, which varies with temperature fluctuations.

### Wind direction and its impact on AQI

**Figure 4:**
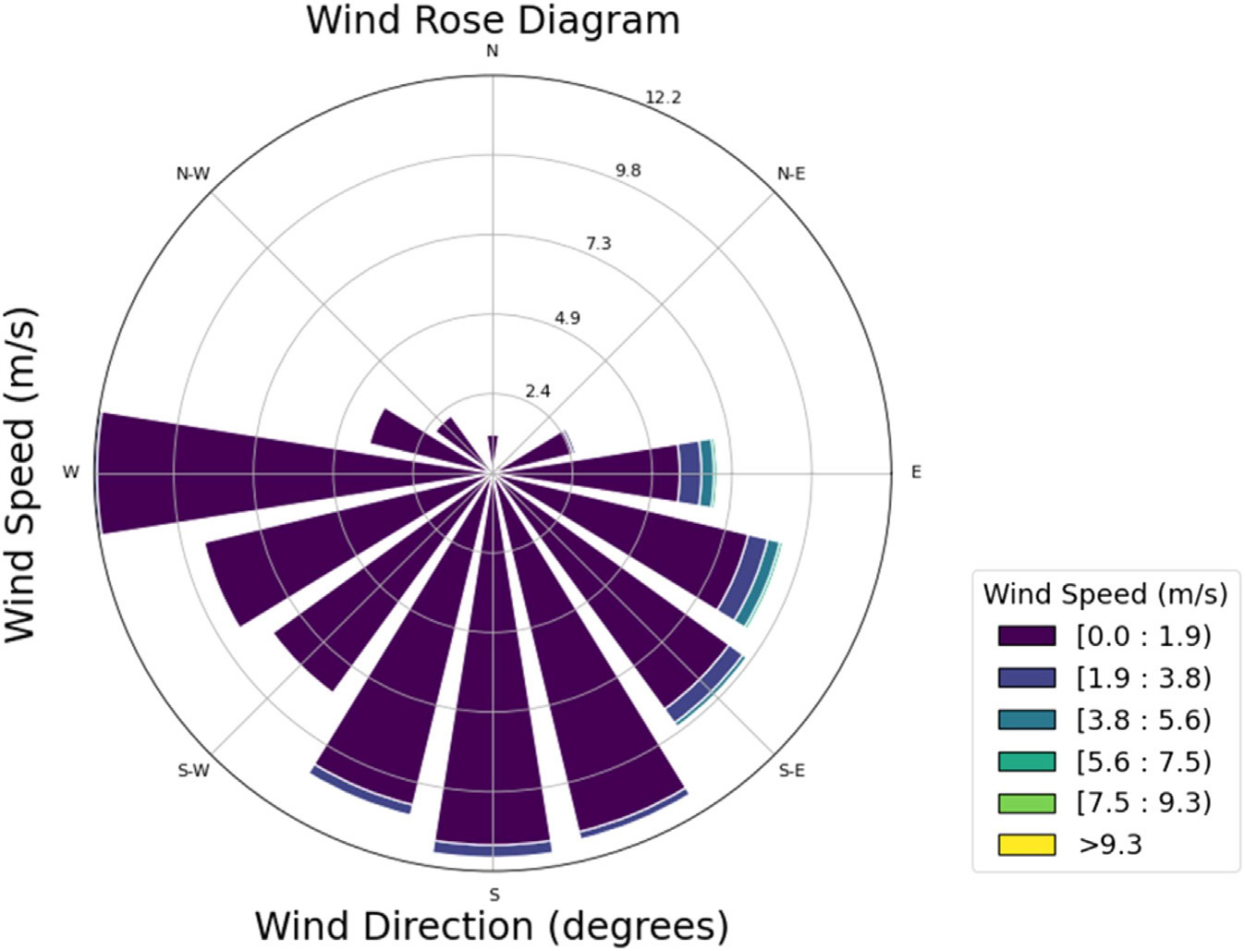
Wind rose diagram of Hyderabad city for the study period of 2016–2023 This graph illustrates the wind rose diagram of Hyderabad city, considering data from 2016 to 2023. Wind speed and wind directions are illustrated to understand the impact of these meteorological parameters on the movement of air pollutants.

The wind rose diagram was used to understand the wind speed and direction of the city, which affects the migration of air pollutants from one place to another.^24^ Figure 4 illustrates Hyderabad’s daily wind rose diagram from 2016 to 2023. Figure 4 shows that the average wind speed in the city was always in the range of 0–1.9 m/s, and, on some days, the average daily wind speed was approximately 1.9–3.8 m/s. The maximum wind speed during the study period was 9.35 m/s. According to the wind rose diagram results, Hyderabad receives 12% wind from the west and 12% from the south. Furthermore, approximately 27% of the wind blows from the south-west and 31% from southeast directions.

Figure S4 illustrates the annual variation of the wind rose diagram from 2016 to 2023, depicting wind speed and direction. Figure S4 shows that, in 2016, the predominant wind direction was from the east and south, with wind speeds varying between 0.3 and 3.9 m/s. In 2017, the wind direction changed slightly from the southeast to the south-west. In most observations, the wind speed exceeded 2.3 m/s. In 2018, the wind direction was primarily from the east, with a maximum speed of 5.8 m/s. There is a border distribution of wind throughout the year. Scattered wind direction was observed in 2019, mainly from the northeast and southeast, with a maximum wind speed of 6.9 m/s. In 2020, the wind speed and direction were similar to those in 2016; the wind speed was much lower than that in 2019. In 2021, wind directions were identical to those in 2017, and the maximum wind speed in a few observations exceeded 3.7 m/s. In 2022, the wind direction is from the east, and the wind speed exceeds 2.2 m/s. In 2023, varying wind directions and speeds were observed, possibly due to atmospheric instability and climate change. The wind direction was uniform in almost all years except 2019 and 2023, when more variation was observed. Similarly, the monthly variation of the wind direction is illustrated in Figure S5.

### Ensemble stacking algorithm performance for AQI prediction

**Figure 5:**
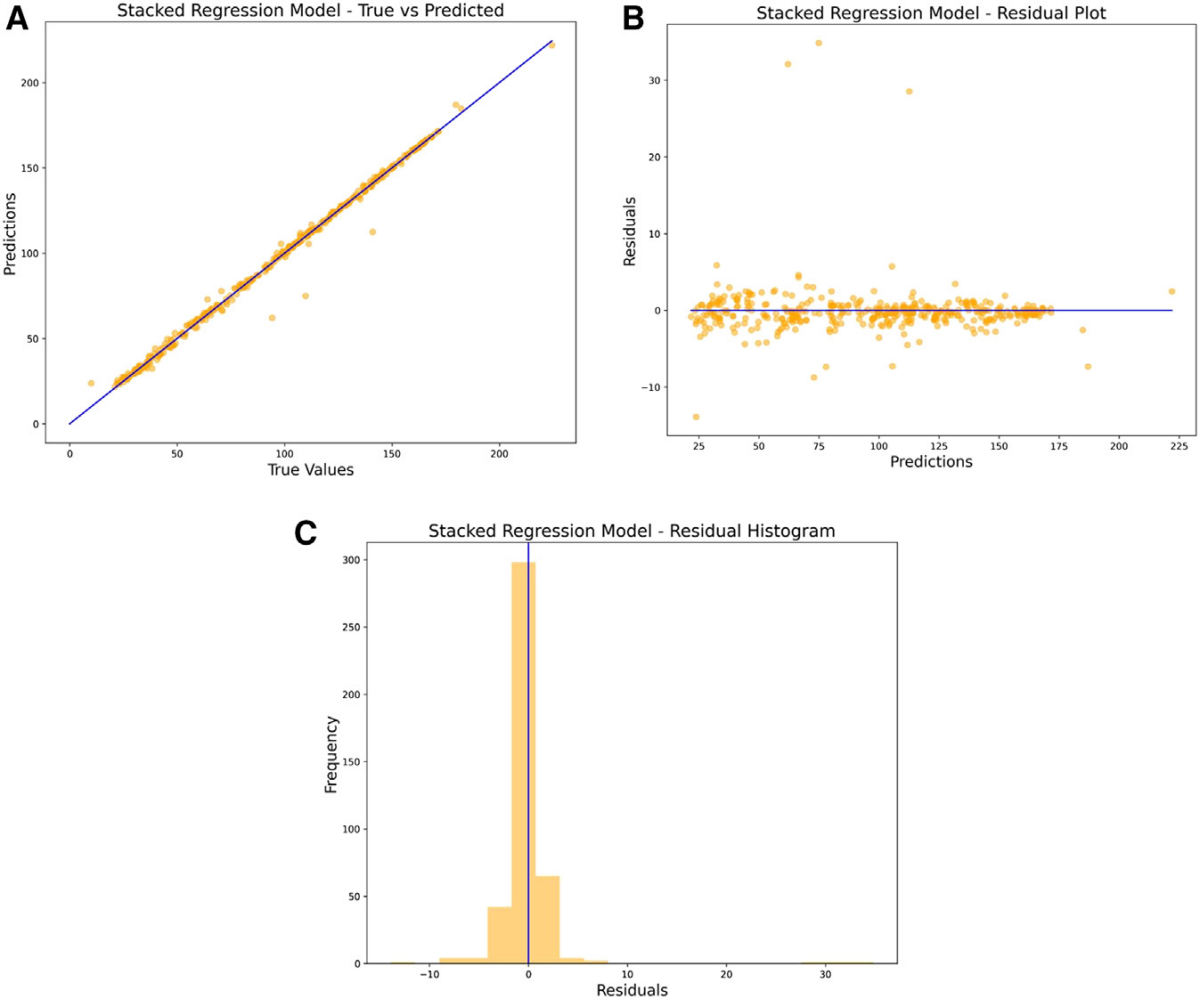
Performance of stacked regression model (A) Scatterplot showing the true values vs. predicted values. (B) Residual plot. (C) Histogram of residuals. The graph is used to evaluate the model performance of the ensemble stacked regression using scatterplots, residual plots, and histogram plots.

**Figure 6:**
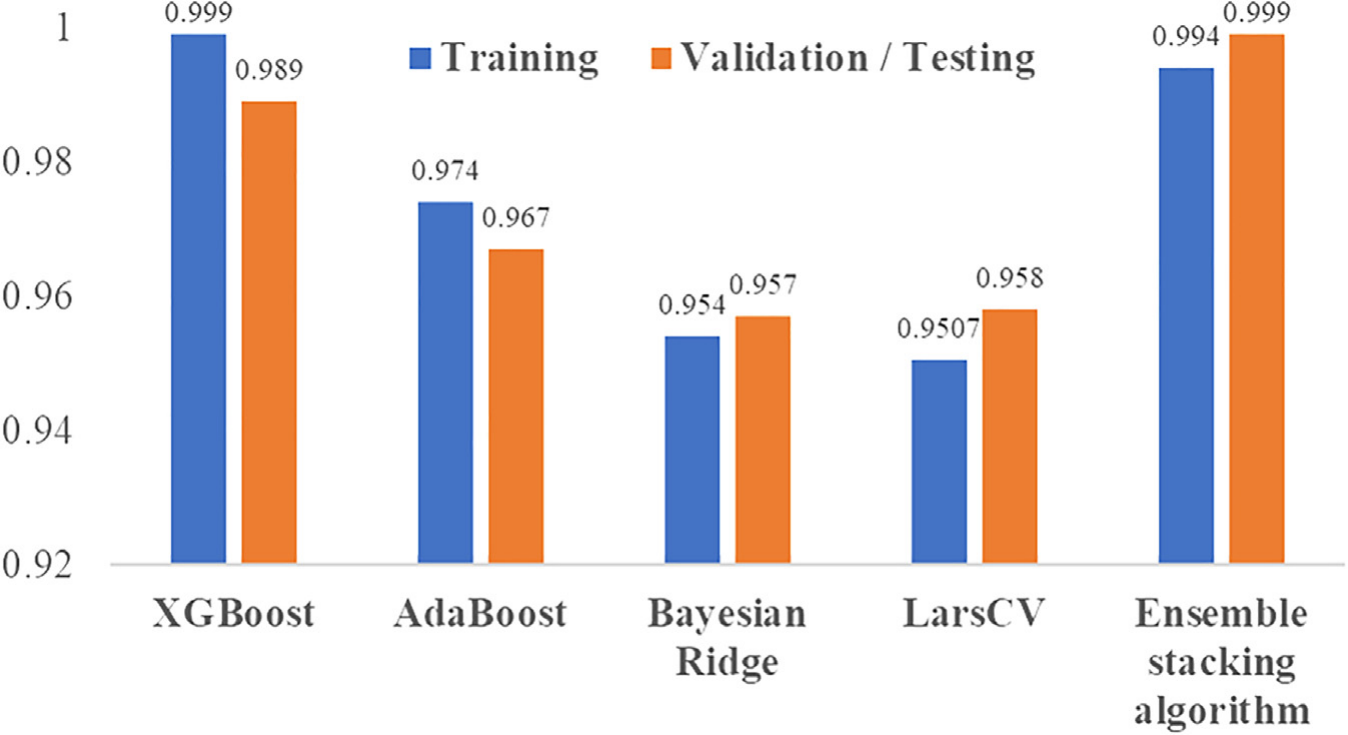
Comparison of performance of different ML models based on R^2^ value This graph illustrates the comparison of different machine learning models with respect to both training and testing datasets. The blue color graph represents the training datasets, and the orange color represents the testing data.

Table 1 presents the optimal hyperparameters for four ML models, namely XGBoost, AdaBoost, Bayesian Ridge, and LarsCV, determined using 5-fold cross-validation via GridSearchCV. These models were fine-tuned to enhance their predictive performance in the AQI prediction task for Hyderabad city. Table 2 provides the performance evaluation of these models for AQI prediction on the training and validation datasets. The ensemble stacking algorithm achieves the best overall performance, with an R^2^ of 0.994 on the training and significantly lower error metrics (mean absolute error [MAE] = 0.496, mean square error [MSE] = 0.429, root-mean-square error [RMSE] = 0.655), demonstrating its superior AQI predictive ability. Compared to training datasets, the ensemble stacking algorithm achieved R^2^ of 0.999 and significantly very few error metrics. The enhanced performance of the ensemble stacking algorithm on the test dataset can be linked to its capability to integrate the advantages of several models, which helps to lessen overfitting to the training data. Moreover, the regularization methods present in the base models or the meta-model might assist in alleviating the impact of model complexity and adjusting more effectively to changes in the dataset distribution. XGBoost demonstrates the best performance among the base models, with a training R^2^ of 0.999 and a validation R^2^ of 0.989, though the errors (MAE = 1.521, MSE = 22.158, RMSE = 4.707) during validation are higher than for other models. AdaBoost performs moderately well with a validation R^2^ of 0.967, but it shows higher error metrics (MAE = 6.058, RMSE = 8.397). Bayesian Ridge and LarsCV perform similarly, with validation R^2^ scores of 0.957 and 0.958, respectively, but they have relatively higher errors than the XGBoost and ensemble stacking algorithm. Figure 5 visually depicts the performance of the ensemble stacking model. The scatterplot in Figure 5A shows the alignment of predicted vs. actual values, indicating excellent model fit as most points lie near the diagonal line. The residual plot in Figure 5B demonstrates that the residuals are centered around zero, indicating no significant bias. The histogram of residuals in Figure 5C shows that the residuals are normally distributed, with most errors concentrated around zero, further validating the model’s reliability. Figure 6 compares the performance of different ML models based on R^2^ value. Table 3 summarizes the comparison of various ML models’ performance in AQI prediction of different cities.

**Table 1:** Optimal hyperparameters for regression models selected via 5-fold cross-validation using GridSearchCV

ML algorithm	Best hyperparameters
XGBoost	‘learning_rate': 0.1, ‘max_depth': 5, ‘n_estimators': 200
AdaBoost	{‘learning_rate': 0.1, ‘loss': ‘exponential', ‘n_estimators': 200}
Bayesian Ridge	{‘alpha_1': 1e-06, ‘alpha_2': 0.0001, ‘lambda_1': 0.0001, ‘lambda_2': 1e-06}
LarsCV	{‘eps': 2.220446049250313e-16, ‘max_n_alphas': 100}

**Table 2:** Comparison of various machine learning models in predicting the AQI for Hyderabad

S. no.	Machine learning model	Training				Validation/testing			
		MAE	MSE	RMSE	R^2^	MAE	MSE	RMSE	R^2^
**Base models performance**									

1	XGBoost	0.321	0.204	0.452	0.999	1.521	22.158	4.707	0.989
2	AdaBoost	6.014	55.398	7.443	0.974	6.058	70.518	8.397	0.967
3	Bayesian Ridge	6.870	98.061	9.902	0.954	6.564	90.574	9.517	0.957
4	LarsCV	7.232	105.80	10.285	0.9507	6.642	89.713	9.471	0.958

**Ensemble model performance**									

5	ensemble stacking algorithm	1.304	10.112	3.180	0.994	0.496	0.429	0.655	0.999

**Figure:**
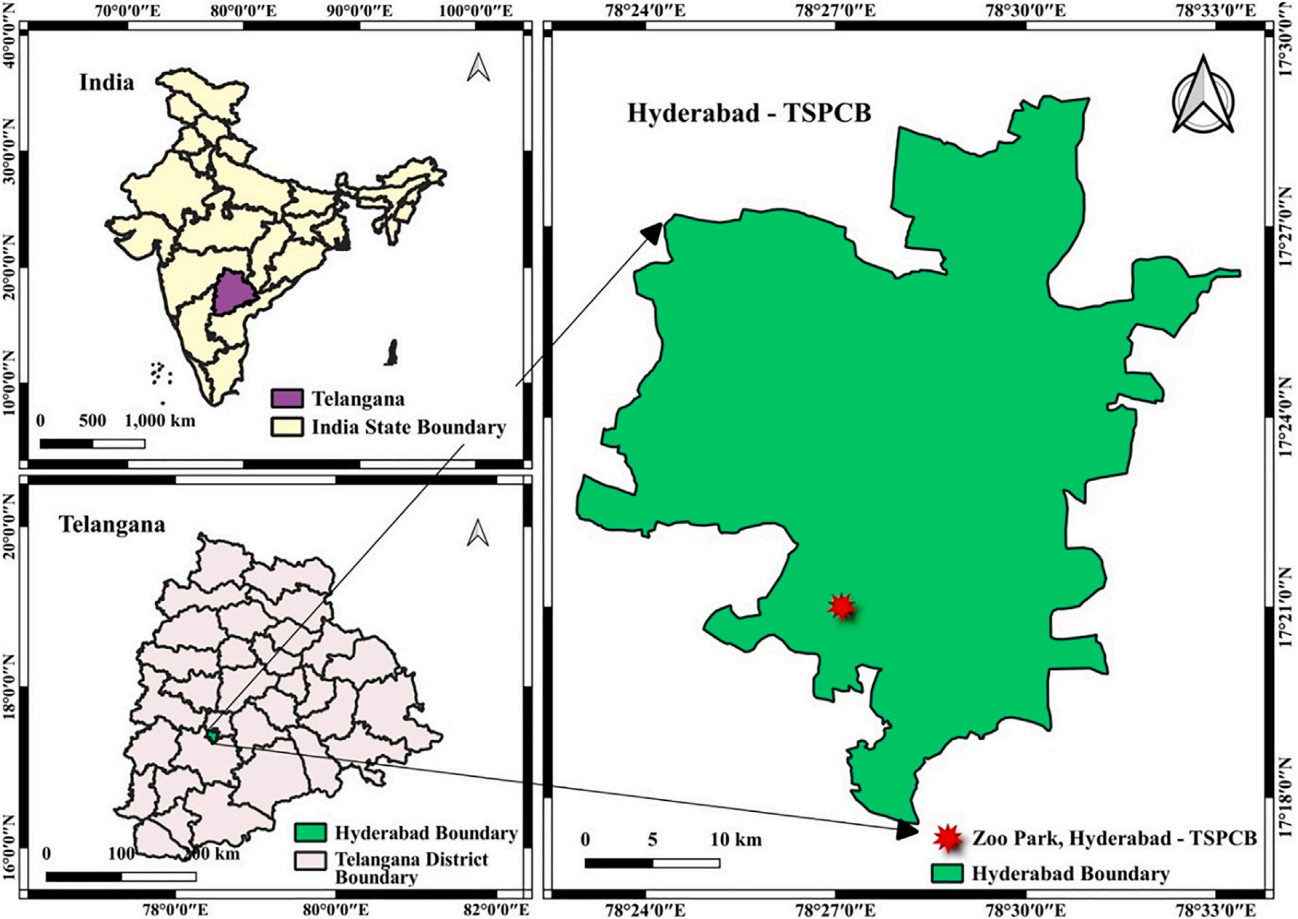
Location map of Hyderabad city, Telangana, and study area The data used in the present study were collected from the location of the Zoological Park station, Hyderabad, having a latitude and longitude of 17.350221 and 78.45155.

**Table 3:** Comparing the performance of various machine learning models in predicting AQI for different cities

City	Machine learning model	R^2^	MAE	MSE	RMSE	MAPE	Reference
Bagdad	random forest	0.977	2.41 × 10^−3^	–	5.35 × 10^−3^	3.67 × 10^−3^	Khadom et al.^27^
	decision tree	0.957	2.08 × 10^−3^	–	7.24 × 10^−3^	4.45 × 10^−3^	
	KNN	0.525	1.55 × 10^−2^	–	2.42 × 10^−2^	1.89 × 10^−2^	
	MLP	0.971	1.49 × 10^−3^	–	2.89 × 10^−3^	2.08 × 10^−3^	
	LSTM	0.983	0.62 × 10^−3^	–	2.15 × 10^−3^	1.21 × 10^−3^	
Chennai	XGBoost	0.9252	0.0857	0.022	0.1501	–	Ravindiran et al.^29^
	random forest (RF)	0.9149	0.0915	0.0256	0.1601	–	
	bagging regressor	0.9167	0.09032	0.0251	0.1584	**-**	
	LightGBM	0.9054	0.0971	0.0285	0.1688	**-**	
Visakhapatnam	LightGBM	0.9915	1.80	27.62	5.25	**-**	Ravindiran et al.^30^
	random forest	0.999	0.41	3.03	1.75	**-**	
	CatBoost	0.9998	0.60	0.58	0.76	**-**	
	AdaBoost	0.9753	7.38	77.79	8.82	**-**	
	XGboost	0.9981	1.48	5.93	2.43		
Shunyi	SVM	0.9215	5.3164	–	9.0609	0.1265	Su et al.^31^
	ELM	0.9207	5.412	–	9.1086	0.1270	
	LSTM	0.9213	5.4423	–	9.0705	0.1288	
	TCN	0.9251	5.4007	–	8.8526	0.1313	
	XGBoost	0.9238	5.4372	–	8.9280	0.1326	
	MLP	0.9265	5.3167	–	8.7641	0.1316	
Wuhan	RF	0.56	21.56	–	28.86	–	Li and Li^32^
	SVM	0.59	20.86	–	27.92	–	
	BP	0.63	19.92	–	26.67	–	
Delhi	LR	0.40	38.86	–	59.22	–	Sarkar et al.^33^
	KNN	0.48	37.86	–	51.63	–	
	SVM	0.58	49.09	–	61.39	–	
	LSTM	0.78	58.97	–	61.37	–	
	GRU	0.66	42.66	–	50.73	–	
Hyderabad	XGBoost	0.989	1.521	22.158	4.707	–	current research
	random forest	0.990	1.291	20.718	4.551	–	
	bagging regressor	0.989	1.367	22.176	4.709	–	
	LightGBM regressor	0.983	2.269	35.862	5.988	–	
	CatBoost	0.987	3.143	23.488	4.846	–	

### Impact of climate change on Hyderabad city

**Figure 7:**
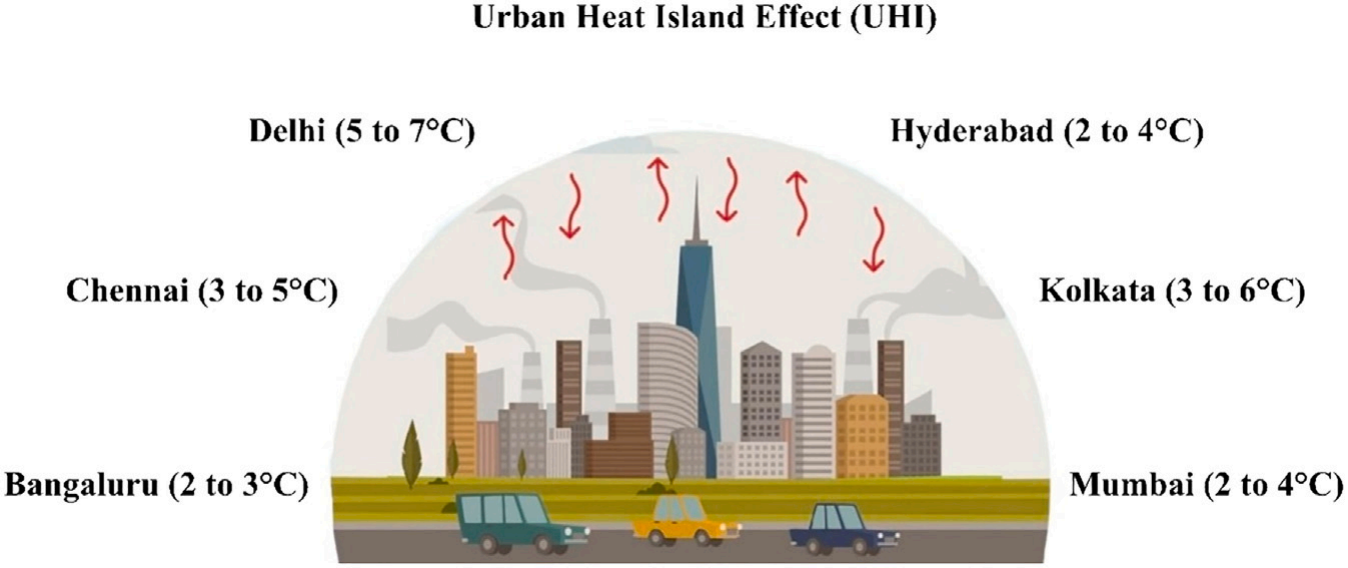
The urban heat island effect of major developed cities in India shows a temperature rise compared to nearby rural areas This information is obtained from the previous studies where the temperature of these cities is higher than that of the surrounding areas.

The impact of air pollutants on climate change is significant, and, as one of the fastest-growing cities, Hyderabad has experienced several environmental issues in recent decades due to particulate matter emissions resulting from urbanization. Air pollutants in Hyderabad have resulted in changes in rainfall patterns, extreme heat waves during summer, urban heat island effects, and air quality degradation.^25^ In 2020, Hyderabad recorded extreme rainfall of 22.4 cm, the highest in the last 130 years. This single-day extreme rainfall event was observed in 2017, 2019, and 2021. Due to heavy rain in 2019, Moti Darwaza of Golconda Fort collapsed, and, in 2020, Majnu Burj of the Golconda Fort and the wall portion of Quila Shahpur subsided. Air pollutants also affect many structures in Hyderabad. Some instances are cracking of wooden surfaces in Naqqar Khana of Badshahi Ashoorkhana, possibly due to high levels of incident solar radiation; tilting of vertical wall surfaces in Sarai Khana of Badshahi Ashoorkhana, possibly due to ground subsidence on one side due to groundwater fluctuations; and cracks on the minaret dome and flaking of plaster on the minaret dome of Toli Masjid, possibly due to high levels of incident solar radiation.^26^ Hyderabad has also experienced the urban heat island effect due to rapid urbanization in the last few years. Hyderabad’s built-up area grew from 234 km^2^ in 2001 to 379 km^2^ (an increase of 161.97%) by 2011 and further expanded to 590 km^2^ (a total increase of 252.14%) by 2021. During the first decade (2001–2011), urbanization led to an average land surface temperature (LST) rise of 0.81°C in the converted areas of Hyderabad, while, in the second decade (2011–2021), the average LST rise was 0.72°C.^27^ Overall, from 2001 to 2021, the average LST of the urbanized areas in Hyderabad increased by 1.3°C. The average urban heat island intensity for Hyderabad over the past two decades was 2.44°C, with an annual increase of 0.033°C/year, 0.030°C/year for the summer season, and 0.044°C/year for the winter season.^28^ Figure 7 illustrates the urban heat island effects of the major developed cities in India. Similar to Bengaluru and Hyderabad, other major cities, namely Delhi, Kolkata, Mumbai, and Chennai, also faced the urban heat island effect.

### Implication and perspectives

Various models, such as XGBoost, AdaBoost, Bayesian Ridge, and LarsCV techniques, provide adaptability in managing different data distributions, noise, and complexity. This variety ensures resilience and applicability in diverse situations. This analysis offers insights into the balance between model precision and computational efficiency. XGBoost, a non-linear and complex model, outperforms linear models such as Bayesian Ridge and LarsCV when dealing with datasets containing intricate relationships. However, XGBoost may encounter overfitting issues if regularization is not properly implemented. Although AdaBoost performs well in many scenarios, it may exhibit a different level of robustness than XGBoost, particularly when handling noisy or extensive datasets. Linear models like Bayesian Ridge and LarsCV have limitations in capturing complex, non-linear relationships, resulting in relatively inferior performance. Stacking, an ensemble technique, delivers the most favorable outcomes, underscoring the effectiveness of combining models to enhance prediction accuracy and generalization capabilities. Furthermore, applying these models to various regions or variables may help to affirm their applicability.

The findings of these models can assist policymakers and public health officials in developing strategies to mitigate air pollution and its health effects, thereby enhancing urban living conditions. Analyzing AQI trends offers valuable insights for shaping policies, but forecasting AQI delivers practical advantages that extend beyond historical data examination. It facilitates proactive measures by predicting days with poor air quality, which allows for timely actions such as implementing traffic restrictions and issuing health warnings. Predictions based on ML can adjust to changing factors like weather conditions and industrial emissions, providing immediate responses and insights during significant events such as smog or dust storms. Furthermore, these forecasts address gaps in monitoring systems, improve public understanding, and aid in scenario planning to assess policy effectiveness. Such forward-thinking capabilities are essential for emergency responses, resource distribution, and long-term strategies that align with sustainable development objectives. Continual model assessment and updating with new data are essential for maintaining accuracy and relevance, particularly in dynamic environments such as air quality monitoring. By effectively leveraging these models, this study contributes to the broader objective of utilizing ML to address intricate environmental issues such as air pollution. However, applicability to other locations requires careful consideration of several factors: variability in pollution sources, climatic and geographic differences, data availability, and quality. Some of the model generalization requirements are data customization, feature adaptation, and transfer learning.

### Conclusion

The present study predicted the AQI based on the data available from 2016 to 2023. The results showed that the pollution levels increased from 2016 to 2019, and, in 2020, pollution levels decreased, possibly due to the nationwide lockdown due to COVID-19. However, the pollutant levels were found to increase the gain from 2021 due to industrial activities, vehicular emissions, and agricultural activities. Further, PM_10_ was found to influence the AQI values significantly, and its correlation coefficient was observed to be 0.96. Other gaseous pollutants were found to have less impact on the AQI, and metrological factors had minimal impact on the AQI predictions. The ML models used in the present study accurately predicted the AQI. The ensemble stacking algorithm demonstrated the highest accuracy, with a correlation coefficient (R^2^) of 0.999 for both the training and validation datasets. XGBoost also performed well, with a correlation coefficient of 0.999 for the training dataset and 0.989 for the validation dataset. This study demonstrates that ML models, particularly ensemble methods, can successfully apply to AQI predictions. However, the scalability of these models to other regions and cities will depend on the quality and availability of localized datasets. The diverse air pollutants and metrological parameters vary, and it will be challenging for the model to apply and assess the transferability of these models to those regions for the prediction of AQI, which needs to be studied.

### Limitations of the study

ML models offer strong and informative forecasts for air quality. However, various constraints must be addressed to enhance accuracy, dependability, and applicability. These limitations include issues with data quality, the risk of overfitting, variations in different regions, and the omission of socioeconomic and policy-related elements. By confronting these obstacles in future studies, the models could evolve into even more effective instruments for comprehending and addressing air pollution, particularly in dynamic settings such as India.

## Resource availability

### Lead contact

Further information and requests for resources should be directed to and will be fulfilled by the lead contact, Azees Maria (azeesmm@gmail.com).

### Materials availability

This study did not generate new unique reagents.

### Data and code availability

• All data reported in this paper are shared in the relevant literature, with DOIs provided in the key resources table under deposited data.
• The source code employed in the current research can be accessed on the GitHub page, and the link is given in the key resources table.
• Additional information for reanalyzing the data reported in this paper is available from the lead contact upon request.

## Acknowledgments

The authors would like to express their gratitude to the Higher Institution Centre of Excellence (HICoE), 10.13039/501100003093Ministry of Higher Education, Malaysia, under the project code 2024001HICOE as referenced in JPT(BPKI)1000/016/018/34(5). This work was supported by 10.13039/100019523Tenaga Nasional Berhad and 10.13039/501100008561Universiti Tenaga Nasional through the BOLD Refresh Postdoctoral Fellowships under the project code of J510050002-IC-6 BOLDREFRESH2025-Centre of Excellence.

## Author contributions

Conceptualization and methodology, G.R. and K.K.; resources, S.R. and D.D.; data curation, B.D. and G.S.; writing – original draft, G.R. and K.K.; writing – review and editing, G.S., B.D., and A.M.; visualization, D.D. and A.M.; supervision, G.H.

## Declaration of interests

The authors declare no competing interests.

## STAR★Methods

### Key resources table

**Table:** 

REAGENT or RESOURCE	SOURCE	IDENTIFIER
**Deposited data**		

Dataset	Central Pollution Board	https://app.cpcbccr.com/ccr/#/caaqm-dashboard-all/caaqm-landing/data

**Software and algorithms**		

Python	Python Software Foundation	https://www.python.org/
Numpy	Harris et al.^34^	https://numpy.org/
Pandas	McKinney et al.^35^	https://pandas.pydata.org/
Matplotlib	Hunter et al.^36^	https://matplotlib.org/
Seaborn	Waskom et al.^37^	https://seaborn.pydata.org/
Scikit-learn	Pedregosa et al.^38^	https://scikit-learn.org/stable/
XGBoost	Chen et al.^39^	https://xgboost.readthedocs.io/
AdaBoostRegressor	Freund et al.^40^	https://scikit-learn.org/stable/modules/generated/sklearn.ensemble.AdaBoostRegressor.html
StackingRegressor	Wolpert et al.^41^	https://scikit-learn.org/stable/modules/generated/sklearn.ensemble.StackingRegressor.html
LarsCV	Hastie et al.^42^	https://scikit-learn.org/stable/modules/generated/sklearn.linear_model.LarsCV.html
Bayesian Ridge Regression	Tipping et al.^43^	https://scikit-learn.org/stable/modules/generated/sklearn.linear_model.BayesianRidge.html
DecisionTreeRegressor	Breiman et al.^44^	https://scikit-learn.org/stable/modules/generated/sklearn.tree.DecisionTreeRegressor.html
Matplotlib.pyplot	Hunter et al.^36^	https://matplotlib.org/stable/api/_as_gen/matplotlib.pyplot.html
DateTime	Python datetime module	https://docs.python.org/3/library/datetime.html
Codes used in the Present Study	Present Study	https://github.com/kkarthiks1/Hyderabad-Air-Quality-Data

### Method details

#### Study area

Hyderabad is the capital of Telangana, India. The city is located on the Deccan Plateau and covers an area of 625 km^2^ at an average elevation of 542 m from the mean sea level. Hyderabad is well known for its cultural heritage, and some of the most important monuments are Golconda Fort, Charminar, and Qutb Shahi Tombs. According to the recent census of India in 2020, Hyderabad has a population of more than 10 million. Hyderabad is also called the “city of pearls” for its early diamond business history of over 400 years. The city is diverse, with different industrial sectors, namely, the IT sector, pharmaceutical industries, and biotechnological research. The city experiences a hot, dry, and tropical wet climate, with hot summers, wet monsoons, and mild winters. March to June is considered summer, and the maximum temperature is approximately 45°C; June to September is considered monsoon season, and temperatures range from 25 to 35°C. October to February is a winter period with a temperature range of 15 to 25°C. April to May is considered the pre-monsoon season, and October to November is the post-monsoon season. The average rainfall is approximately 750 mm, and the city receives maximum rainfall from the south-west monsoon between June and September. Figure 8 illustrates a map of the study area.

#### Feature selection for the prediction of AQI

The datasets used in this research were downloaded from the Central Pollution Board – Central Control Room for Air (CPCBCRR- https://app.cpcbccr.com/ccr/#/caaqm-dashboard-all/caaqm-landing/data). The data used for the study were collected from the Zoological Park Station, Hyderabad, located at a latitude and longitude of 17.350221 and 78.451558, respectively (Data & Code Link: https://github.com/kkarthiks1/Hyderabad-Air-Quality-Data**).** For the prediction of AQI, both air quality and meteorological parameter data were used. The air quality data used are Particulate matter 2.5 and 10, (PM_2.5_ (μg/m^3^), PM_10_ (μg/m^3^)), Nitric oxide (NO (μg/m^3^)), Nitrogen dioxide (NO_2_ (μg/m^3^)), Nitrogen oxides (NOx (ppb)), Ammonia (NH_3_ (μg/m^3^)), Sulphur dioxide (SO_2_ (μg/m^3^)), Carbon monoxide (CO (mg/m^3^)), Ozone (O_3_ (μg/m^3^)), Xylene (μg/m^3^), and Benzene (μg/m^3^). Meteorological parameters included Wind Direction (WD (degree)), Solar Radiation (SR (W/mt^2^)), Barometric Pressure (BP (mmHg)), vertical wind speed (VWS (m/s)), Ambient Temperature (AT (degree C)), rainfall (RF (mm)), and TOT-RF (mm). AQI was considered the target variable in the present study. The data used in the present study were collected from 09^th^ August 9, 2016, to 27^th^ October 27, 2023, with a total observation of 2636 (daily average data). The overall process involved in the prediction of AQI begins with data preprocessing steps, including missing value handling, data analysis, and Box-Cox transformation. The dataset is then split into training and test sets. After hyperparameter tuning using GridSearchCV with 5-fold cross-validation, the best hyperparameters for XGBoost, LarsCV, Bayesian Ridge, and AdaBoost models are determined. These models are then combined in an ensemble stacking approach, with Bayesian Ridge serving as the meta-model to produce the final AQI prediction.

#### Ensemble Stacking Algorithm

In this research, an ensemble stacking algorithm combining multiple regression models (XGBoost, Lasso Least Angle Regression (LarsCV), Bayesian Ridge, and AdaBoost) was used, with their best hyperparameters found via GridSearchCV. Stacking is an ensemble learning technique that combines multiple base models to improve predictive performance. Instead of using a simple average or majority vote, as in bagging or boosting, stacking uses a meta-model (a secondary model) that learns from the outputs of the base models to produce a final prediction. The idea is to leverage the strengths of different algorithms to create a model that performs better than any individual model.

XGBoost, LarsCV, Bayesian Ridge, and AdaBoost are used as base learners. XGBoost trains multiple weak learners (decision trees) sequentially, where each new learner corrects the errors of the previous ones. LarsCV iteratively builds a regression model by including features that are most correlated with the residuals. Bayesian Ridge optimises the weights by considering the uncertainty in the model's parameters and regularizing the model by adding penalty terms to the likelihood function to avoid overfitting. AdaBoost sequentially trains weak learners (usually decision trees), with each subsequent model focusing more on instances where the previous model made errors.

Bayesian Ridge is used as the meta-model (final estimator), which learns from the predictions made by the base models to make a final prediction. The predictions from the base models (XGBoost, LarsCV, Bayesian Ridge, and AdaBoost) are treated as input features for the Bayesian Ridge model. Bayesian Ridge adds a probabilistic framework to linear regression, which is useful for handling uncertainty and variance in the predictions from the base models. A 5-fold cross-validation strategy is employed to make the ensemble more robust and avoid overfitting. The stacking process involves training each base model on the training data, collecting the predictions of each base model on the validation sets during cross-validation, and using these predictions as inputs to the meta-model, which then learns to combine the base models' outputs to generate the best possible predictions.

#### Pre-processing of datasets

2636 observations were obtained from the CPCBCRR for the above duration for 23 parameters, including AQI. In all the datasets, missing values may arise due to malfunction in the sensors, errors during data transmission, and issues with data collection. These will result in data errors and reduce the accuracy of the model. Missing values in the dataset must be removed before processing. The missing values in all 23 parameters were removed, and the final imputed dataset consisted of 2113 observations. The AQI category distribution was made using the 2113 observations, which showed that 1153 observations were moderate, 537 observations were satisfactory, 408 observations were good, 13 observations were poor, and two observations were very poor. Figure S1 shows the AQI categories based on these datasets. The AQI underwent a conversion from object type to float type. Statistical analysis of the air pollutant and meteorological parameter datasets is summarised in Table 4. The available data was split into training (80% of datasets) and testing (20% of the datasets) to develop the Ensemble Stacking Algorithm for AQI Prediction. The performance and accuracy of the different ML models in the prediction of the AQI were analysed using various types of errors, namely the root mean square error (RMSE), Mean Absolute Error (MAE), Mean Square Error (MSE) and R^2^ (Correlation coefficient).

**Table:** Statistical analysis of the datasets used in AQI prediction

Features	Count	Mean	Std	Min	25%	50%	75%	Max
PM_2.5_ (μg/m^3^)	2,113	48.11	23.62	3.21	25.15	50.3	69.74	126.18
PM_10_ (μg/m^3^)	2,113	101.36	49.91	4	56.17	104.79	145.36	241
NO (μg/m^3^)	2,113	8.00	6.32	0.14	2.68	6.62	10.81	45.65
NO_2_ (μg/m^3^)	2,113	37.13	22.30	0.17	17.98	34.52	50.35	130.59
NOx (ppb)	2,113	25.98	14.65	0.04	13.26	24.5	35.94	102.6
NH_3_ (μg/m^3^)	2,113	10.89	8.15	0.11	3.42	10.24	16.57	79.71
SO_2_ (μg/m^3^)	2,113	5.97	6.72	0.15	2.69	3.86	5.13	77.84
CO (mg/m^3^)	2,113	0.78	0.44	0	0.46	0.78	1.04	8.43
Ozone (μg/m^3^)	2,113	28.29	18.53	4.32	14.85	21.32	36.11	127.92
Benzene (μg/m^3^)	2,113	2.07	2.38	0	0.47	1.25	2.94	20.32
WD (degree)	2,113	189.76	66.50	3	138.25	186.72	246.91	360
SR (W/mt^2^)	2,113	121.55	84.01	1.54	80.94	111.56	145.79	1,065.51
BP (mmHg)	2,113	714.37	23.64	700.88	708.03	711.05	715.77	1,054.96
VWS (m/s)	2,113	−2.02	2.03	−14.84	−3.8	−1.34	−0.2	16.51
AT (degree C)	2,113	26.52	3.40	0.92	24.34	26.2	28.24	41.73
RF (mm)	2,113	0.41	1.76	0	0	0	0.1	22.5
Xylene (μg/m^3^)	2,113	2.99	7.26	0	0.23	0.92	2.14	52.78
TOT-RF (mm)	2,113	34.09	143.52	0	0	0	10	2,070
AQI	2,113	101.58	46.39	9.98	61.94	106.05	139.95	304.75

#### Exploratory data analysis

Exploratory data analysis (EDA) is a preliminary step in understanding the key characteristics and relationships between different variables/features used in the datasets. It is generally used to understand the dataset distributions and their ranges, central tendencies, and distributions. EDA can detect outliers, handle missing values, and calculate key statistics.^45^ A correlogram is a heatmap variant used to find the correlation between each variable, and other variables spread over two axes. Figure 9 Illustrates a heatmap of the variables used in the datasets, demonstrating the variables that significantly impact the AQI prediction. A correlation greater than 0.5 is considered as threshold, and a correlation is classified as positive or negative. The correlation coefficient varies from -1 to +1, and a correlation close to 1 indicates a perfect fit of the model to the actual data. The Pearson correlation coefficient was developed to understand the relationship between the two features.^46^ Figure 9 shows that PM_2.5_ and PM_10_ have a more significant correlation of 0.95 and 0.96, respectively, with AQI. Metrological parameters have minimal impact on AQI compared with air pollutants.

**Figure:**
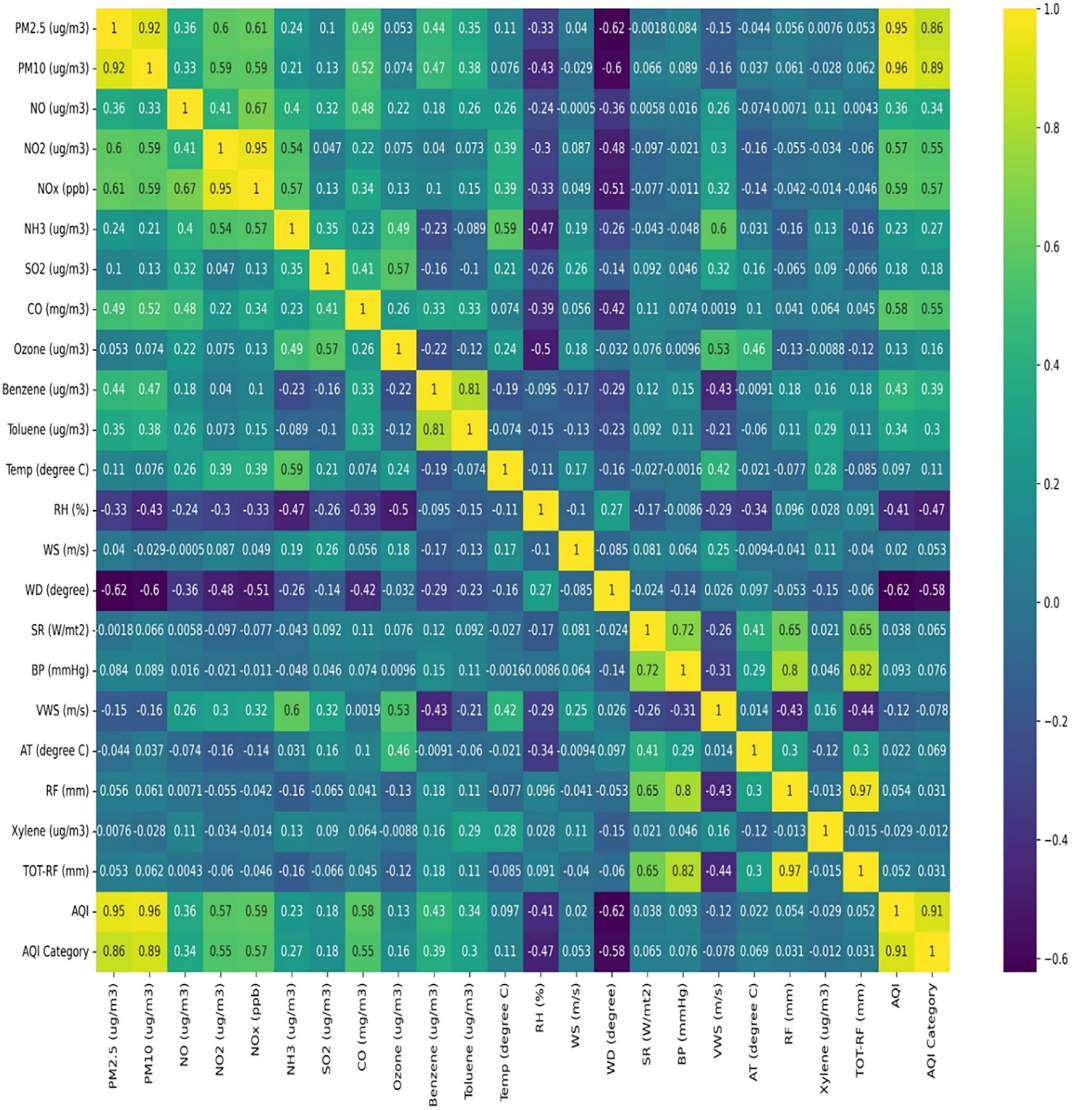
Heatmap-correlation analysis of different features used in AQI predictions This image illustrates the correlation of each variable used in the data with other variables to understand the impact.

#### Data transformation

The skewness and kurtosis of the datasets with various features are outlined in Table 5, both before and after Box-Cox transformation (BCT). Skewness and kurtosis were used to analyse other features and how much they deviated from the normal distribution. Skewness was used to measure the symmetry of the distribution of the various features.^47^ Generally, skewness ranges between -1 and +1, which is excellent; -2 to 2 is acceptable, and values greater than -2 to +2 suggest that the distribution is abnormal. Kurtosis analyses datasets that are too peak or flat compared with the normal distribution. The peak distribution is called positive kurtosis, and the flat distribution is called negative kurtosis.^48^ A kurtosis greater than +2 is called positive kurtosis, and less than -2 is called negative kurtosis. The dataset was found to be normally distributed when skewness and kurtosis were close to zero. From Table 5, it was observed that features, namely NO, SO_2_, CO, Ozone, Benzene, Toluene, WS, SR, BP, RF, Xylene and TOT-RF, had skewness and kurtosis of more than +1, indicating that the features were not normally distributed. Some parameters, namely PM_2.5_, PM_10_, NO_2_, NOx, NH_3_ and RH, have less skewness and kurtosis, indicating that the data are typically distributed. BCT was applied to make the feature a normal distribution, and the results showed that skewness and kurtosis were reduced and better suited for further data analysis. BCT is a statistical technique used to reduce the raw datasets' variance, skewness, and kurtosis and convert them into normal distribution. The significance of the BCT is to achieve high accuracy using the ML algorithm. If BCT is not used, accuracy and bias in AQI predictions will be reduced. Applying BCT in the present study enhanced the data scaling, and data was distributed normally.

**Table:** Data transformation (before and after) of skewness and kurtosis for the datasets

Parameters	Before data transformation		After data transformation	
	Skew	Kurtosis	Skew	Kurtosis
PM_2.5_ (μg/m^3^)	−0.095	−1.218	−0.095	−1.218
PM_10_ (μg/m^3^)	−0.086	−1.173	−0.086	−1.173
NO (μg/m^3^)	1.724	4.423	0.003	−0.608
NO_2_ (μg/m^3^)	0.764	0.329	0.764	0.329
NOx (ppb)	0.714	0.391	0.714	0.391
NH_3_ (μg/m^3^)	0.776	1.657	0.776	1.657
SO_2_ (μg/m^3^)	3.252	15.172	0.003	0.326
CO (μg/m^3^)	4.224	62.401	−0.009	0.037
Ozone (μg/m^3^)	1.879	4.036	0.069	−0.456
Benzene (μg/m^3^)	2.407	8.925	0.090	−1.011
Toluene (μg/m^3^)	3.457	16.783	−0.012	−0.511
Temp (degree C)	0.414	17.607	0.414	17.607
RH (%)	−0.086	0.142	−0.086	0.142
WS (m/s)	4.395	29.145	0.315	−1.023
WD (degree)	0.098	−0.726	0.098	−0.726
SR (W/mt^2^)	4.693	37.668	0.225	4.112
BP (mmHg)	9.504	106.892	0.000	0.000
VWS (m/s)	−0.416	4.648	−0.416	4.648
AT (degree C)	0.087	4.826	0.087	4.826
RF (mm)	7.442	65.939	1.338	0.175
Xylene (μg/m^3^)	4.305	19.482	0.213	−0.876
TOT-RF (mm)	7.980	79.797	0.791	−1.271
AQI	−0.002	−0.719	−0.002	−0.719

#### Data splitting and composition

The dataset was organised into features (X) and target variables (y), excluding the 'Date' and 'AQI' columns. It was divided into training and test sets at an 80:20 ratio. The present research used a training set of 1690 samples to train the machine learning models and a test set of 423 samples to evaluate their performance. Both sets contain 22 features, excluding the 'Date' and 'AQI' features.

#### GridSearchCV-based hyperparameter tuning

Hyperparameter tuning for the base models—XGBoost, Lasso Least Angle Regression (LarsCV), Bayesian Ridge, and AdaBoost—was performed using GridSearchCV with 5-fold cross-validation. Initially, the hyperparameters to be tuned for the machine learning algorithms were defined. GridSearchCV systematically searches through combinations of hyperparameter values specified in the parameter grid to find the optimal configuration that maximises model performance. The model's performance is evaluated using the R^2^ score, which measures how well the model explains the variance in the target variable. A 5-fold cross-validation strategy was employed to identify the best hyperparameters. The data was split into five subsets, each serving as a validation set while the remaining folds were used for training. Cross-validation ensures that the model's performance is evaluated across multiple data splits, making it more robust to overfitting and less dependent on a specific data partition.

### Quantification and statistical analysis

Table 4 summarises the statistical analysis of the features used in AQI prediction. The standard deviation was very high for PM_2.5_, PM_10_, WD, SR, BP, and TOT-RF, indicating more fluctuation and variation in the datasets compared to the other features with fewer standard deviations. A lower standard deviation indicates that the dataset values are close to the mean values, and a high standard deviation indicates the variability of the data.^49^ The 1^st^ quartile, median and 3^rd^ quartiles are the key statistics used to understand the distribution of the datasets. It also identifies the central tendency, variability, and skewness of data. The 1^st^ quartile or 25^th^ percentile, or Q1, denotes the lower end of the distribution.^50^ For example, PM_2.5_ had for 25% of the data less than 25.15 μg/m^3^. Similarly, 25% of the data was less than 56.17 μg/m^3^ for PM_10_. The median or 50^th^ percentile or Q2 indicates the data's midpoint; for instance, 50% of the PM_2.5_ is less than 50.3 μg/m^3^. The 3^rd^ quartile, or 75^th^ percentile, represents the upper bound of the data, and indicates the higher end of the data. For instance, 75% of the PM_2.5_ are less than 69.74 μg/m^3^. In addition to these quartiles, interquartile ranges were used to find the spread of datasets close to the middle 50% to identify the variability that was not included by outliers. For instance, the PM_2.5_ interquartile was calculated as the difference between Q3 and Q1 and was observed as 44.59 μg/m^3^ which is close to the median of PM_2.5._

### Additional resources

No additional resources were generated or used.
