# Supplementary material for: Ensemble stacking of machine learning models for air quality prediction for Hyderabad city in India
Source: iScience. 2025 Jan 25;28(2):111894. doi: 10.1016/j.isci.2025.111894 (PMC11883379; doi:10.1016/j.isci.2025.111894)
Supplement: Document S1. Figures S1–S5 and Table S1 [file mmc1.pdf]

iScience, Volume *28*

Supplemental information

Ensemble stacking of machine learning models for air quality prediction for Hyderabad city in India

Gokulan Ravindiran, K. Karthick, Sivarethinamohan Rajamanickam, Deepshikha Datta, Bimal Das, G. Shyamala, Gasim Hayder, and Azees Maria

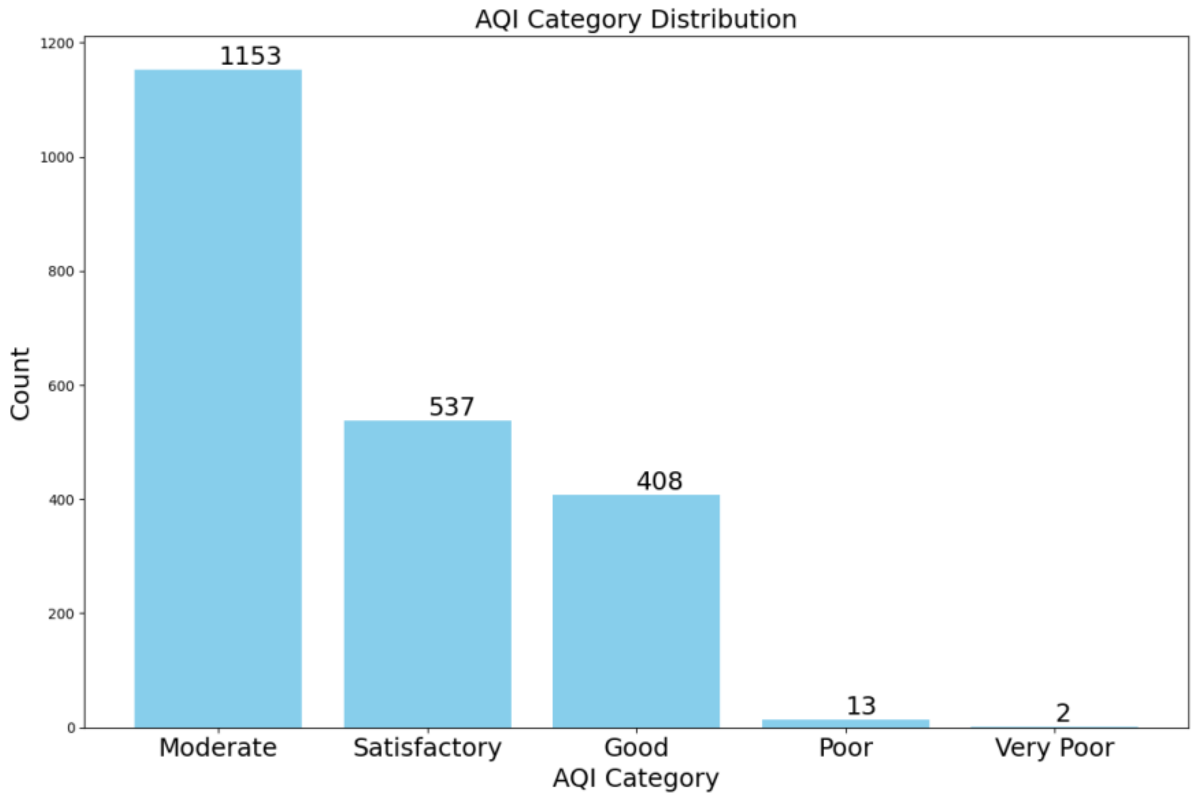

Fig. S1: AQI Classification based on the datasets available for Hyderabad.

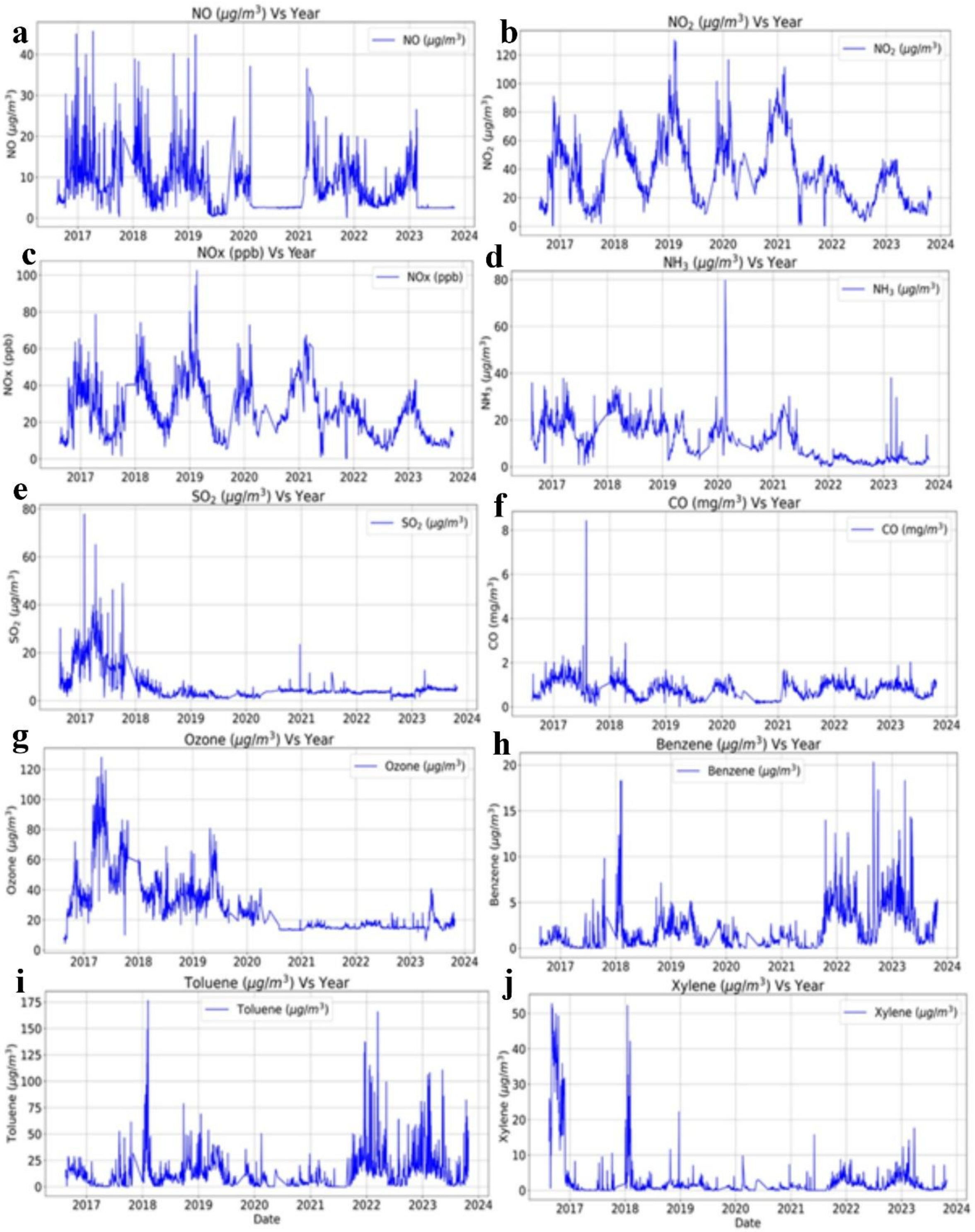

Fig. S2. Seasonal variation of Gaseous Pollutants ((a) NO; (b) NO_2; (c) NO_X; (d) NH_3; (e) SO_2; (f) CO; (g) Ozone; (h) Benene; (i) Toluene; (j) Xylene) from 2016 to 2023.

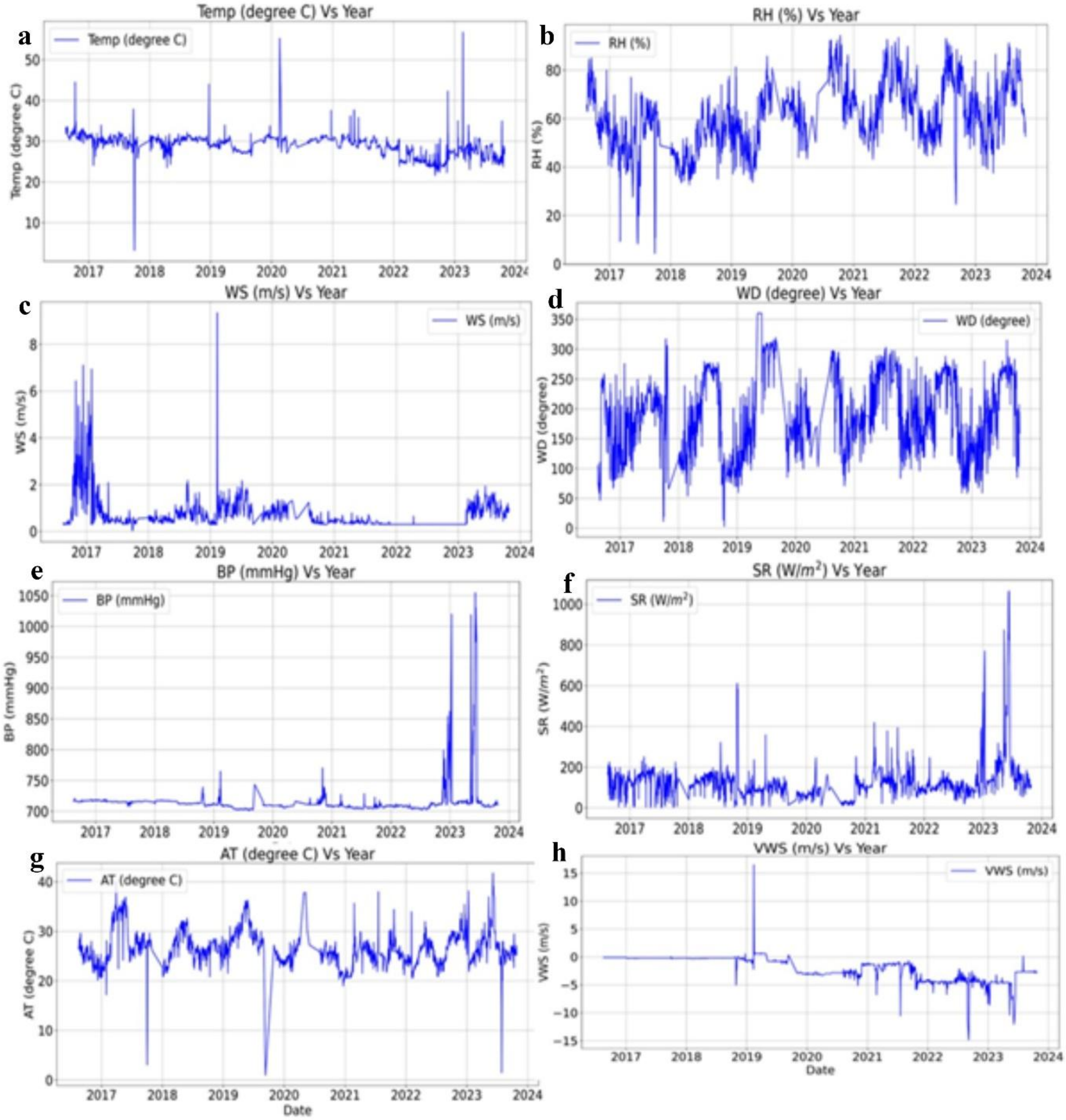

Fig. S3. Seasonal Variation of Meteorological Factors ((a) Temp; (b) RH; (c) WS; (d) WD; (e) BP; (f) SR; (g) AT; (h) VWS) from 2016 to 2023.

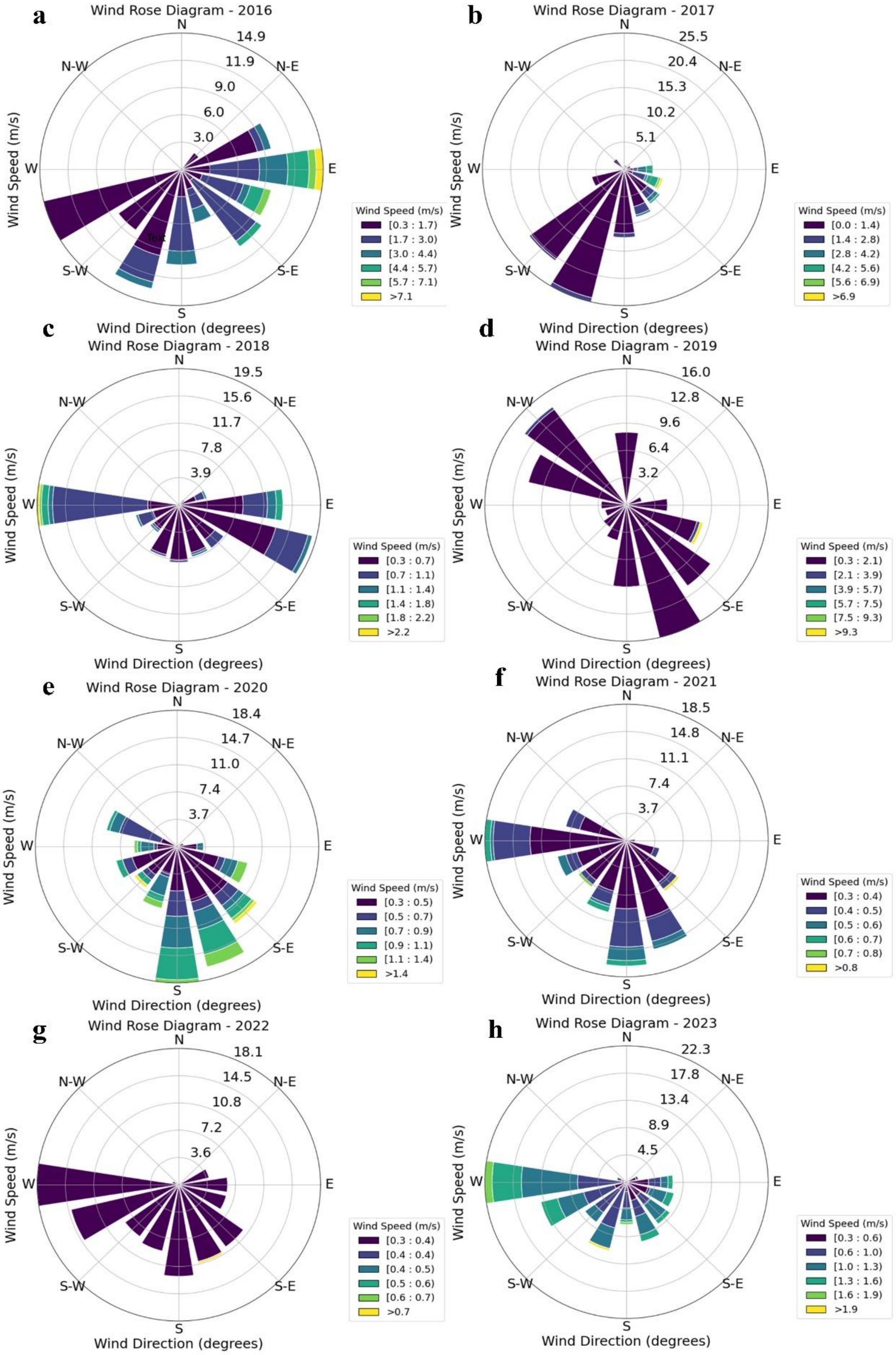

Fig. S4. Wind Rose diagram of Hyderabad city based on annual variations ((a) 2016; (b) 2017; (c) 2018; (d) 2019; (e) 2020; (f) 2021; (g) 2022; (h) 2023).

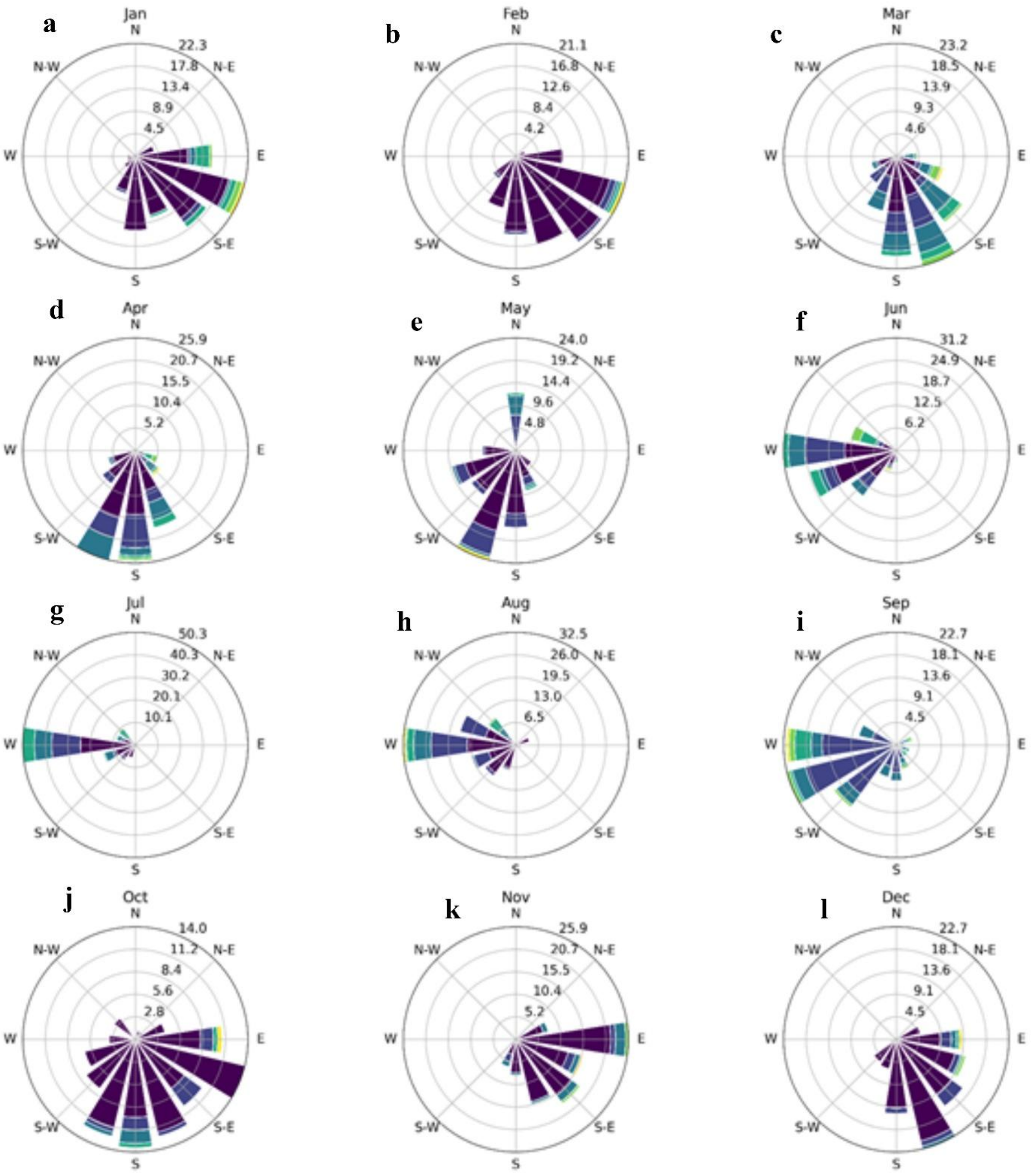

Fig. S5: Seasonal variation of wind rose ((a) Jan; (b) Feb; (c) March; (d) April; (e) May; (f) June; (g) July; (h) August; (i) September ; (j) October; (k) November; (l) December)

Table S1. AQI Ranges for different air pollutants. (https://cpcb.nic.in/displaypdf.php?id=bmF0aW9uYWwtYWlyLXF1YWxpdHktaW5kZXgvQWJvdXRfQVFJLnBkZg==)

AQI Category	PM_{10} 24 hr	$PM_{2.5}$ 24 hr	NO_2 24 hr	O_3 24 hr	CO 8 hr	SO_2 24 hr	NH_3 24 hr	Pb 24 hr	Health Impact
**Good (0-50)**	0-50	0-30	0-40	0-50	0-1	0-40	0-200	0-0.5	Minimal Impact
**Satisfactory (51-100)**	51-100	31-60	41-80	51-100	1.1-2.0	41-80	201-400	0.5-1.0	It may cause minor health problems for sensitive people
**Moderate (101-200)**	101-250	61-90	81-180	101-168	2.1-10	81-380	401-800	1.1-2.0	People with lung diseases, infants and older people will feel discomfort while breathing
**Poor (201-300)**	251-350	91-120	181-280	169-208	10.1-17	381-800	801-1200	2.1-3.0	Causes discomfort to people with respiratory and heart disease
**Very Poor (301-400)**	351-430	121-250	281-400	209-748	17.1-34	801-1600	1201-1800	3.1-3.5	It may cause respiratory problems during prolonged exposure.
**Severe (401-500)**	430+	250+	400+	748+	34+	1600+	1800+	3.5+	Even healthy people will get respiratory problems, and serious health impacts will be there on people with heart and lung diseases.
